# Understanding the Motivation to Write Reviews for Mobile Apps among German Users: Testing the Expanded Theory of Planned Behavior Using a Structural Equation Modeling Approach

**DOI:** 10.1007/s41347-018-0063-5

**Published:** 2018-06-26

**Authors:** Ardion Beldad, Charalampos Voutsas

**Affiliations:** Faculty of Behavioural, Management, and Social Sciences, Department of Communication Science, University of Twente, P.O. Box 217, 7500 AE Enschede, The Netherlands

**Keywords:** Mobile app reviews, Theory of planned behavior, Function of review writing, Writing review intention

## Abstract

Online reviews can make or break a mobile app. Various studies have shown that reviews, especially when their valence is primarily positive, can contribute to the survival of a particular app in a stiffly competitive market. Hence, it is in the best interest of app developers to gain insights into the motivations of app users to review apps they have used. To address the question on the factors influencing people’s intention to write reviews for apps, an extended version of the Theory of Planned Behavior (with the inclusion of various writing functions as predictors) was tested with data from 203 German mobile app users. Results of structural equation modeling reveal that people’s intention to review certain apps is a function of four factors, namely their attitude towards writing reviews, subjective norm, and review writing’s ego-defensive and emotional expression functions. Furthermore, both review writing’s ego-defensive and emotional expression functions positively influence app users’ attitude towards writing reviews.

## Introduction

Online reviews benefit not only consumers but also sellers. For customers devoid of prior experience with a certain product or service, other customers’ narratives about their experience with a product or experience would certainly suffice as a relevant information source. One point that reverberates in the literature on online reviews is the critical role those reviews play in shaping customers’ purchase decisions.

Online reviews, specifically when their valence is positive, have been reported to significantly shape customers’ attitude towards the reviewed product (Ballantine and Yeung 2015) and their willingness to purchase a product (Chevalier and Mayzlin 2006; Lin et al. 2011) or subscribe to a service (Sparks & Browning, 2011; Vermeulen and Seegers 2009). For commercial organizations, online reviews are a new element in marketing communication mix and can perform the role of salespersons, as reviews help customers in identifying products that perfectly suit their needs and conditions (Chen and Xie 2008). The usefulness of reviews for customers is grounded on the notion that users associate reviews with recommendations from real people (Kuehnhausen and Frost 2013).

Just as products and services available in the online market are constantly subjected to online customer reviews, mobile apps, as new commodities, have increasingly become targets for user assessment or evaluation. Reviews of mobile apps enable users to promote an app they are satisfied with or to send warnings of an app’s limitations to potential users (Vasa et al. 2012). For people without any experience with an app, reviews about it are the sole bases for users’ decision to download that app, as those reviews are valuable cues for assessing the app’s quality (Burgers et al. 2016; Kuehnhausen and Frost 2013) and desirability (Burgers et al. 2016).

One would be hard pressed to dispute the indispensability of reviews for a certain mobile app, as previous research has shown that the high-volume and high-user review scores can partly contribute to an app’s sustainability (Lee and Raghu 2014). Additionally, increased sales of mobile apps (Liang et al. 2016) and likelihood of app downloads (Burgers et al. 2016; Huang and Bashir 2017) have been attributed to online reviews. Furthermore, positive comments on the product and the service quality of an app have been reported to increase sales of that app (Liang et al. 2016).

Empirical studies into reviews of mobile apps are gaining momentum considering the increasing popularity of mobile apps. For instance, researchers have looked into what users emphasized (Liang et al. 2016) and what they complain about (Iacob et al. 2013; Khalid et al. 2015) when reviewing apps, the length of mobile app reviews (Vasa et al. 2012), and the impact of certain review elements (e.g., valence, sidedness) on people’s attitude towards an app during a trial period (Huang and Korfiatis 2015). A content analysis of reviews for bipolar disorder apps focused on the themes highlighted (e.g., benefits of the app, privacy and technical issues) in those reviews (Nicholas et al. 2017). However, the underlying mechanism behind people’s decision to write reviews for mobile apps still remains to be thoroughly understood.

Although the factors influencing customers’ inclination to write products and services purchased online have been previously identified (e.g., Dellarocas et al. 2010; Picazo-Vela et al. 2010), research into the predictors of mobile app review writing intention remains scant. Given substantial differences between mobile apps and physical products (e.g., some apps can be downloaded for free, while products bought online need to be paid), variations in the factors influencing individual decisions to review either a physical product or a mobile app might exist.

The current research’s primary goal of determining the factors influencing mobile app review writing intention is realized by testing Ajzen’s (1991) Theory of Planned Behavior (TPB) and by expanding with the inclusion of review writing functions (utilitarian, social, ego-defensive, emotional expression) as possible predictors of people’s attitude towards review writing, based on an initial model proposed to understand people’s motivation for creating user-generated contents (Daugherty et al. 2008). The current study is predicated on these three central questions:

1. To what extent do the TPB factors—attitude, perceived behavioral control, and subjective norm—influence users’ willingness to write reviews for mobile apps?
2. To what extent do the utilitarian, social, ego-defensive, and emotional expression functions of review writing influence users’ willingness to write reviews for mobile apps?
3. To what extent do the utilitarian, social, ego-defensive, and emotional expression functions of review writing influence app users’ attitude towards writing reviews for mobile apps?

## Theoretical Framework

### The Theory of Planned Behavior

While the determinants of people’s willingness to write online reviews for products or services have already been identified in previous studies (Hennig-Thurau et al. 2004; Yoo and Ulrike 2008), the status of mobile apps as recently introduced commodities in the online market, unlike more established products (e.g., compact discs, books) and services (e.g., restaurants, hotels), signifies that the mechanism behind users’ inclination to review certain apps is still insufficiently understood. More importantly, as mobile apps can be enjoyed for free or after paying a certain amount for them, it is likely that people will have different considerations when deciding whether or not to review an app, especially if the app did not cost them a cent.

Given the research’s focus on behavioral intention, specifically the intention to review an app, the Theory of Planned Behavior (TPB) will be primarily employed to gain insight into the predictors of app users’ inclination to review an app for public consumption. Additionally, the centrality of TPB in studies into the factors influencing customers’ intention to review online products (e.g., Picazo-Vela et al. 2010) and the determinants of online collaboration and knowledge sharing (Cho et al. 2010) justifies the appropriateness of the theory as a basis for understanding mobile app review writing intention. Moreover, given the highly rational nature of review writing intention (e.g., cost-benefit calculation prior to the decision to post a review; Cheung and Lee 2012), a theory that primarily considers the rational bases for human behavior and behavioral intention (as primarily exemplified by TPB) should undoubtedly be relevant.

As a modified version of the Theory of Reasoned Action (TRA), TPB postulates that people’s actual performance of a certain behavior is a function of their intention to perform that behavior, which, subsequently, are predicated on three factors, namely, attitude towards the behavior, subjective norm, and perceived behavioral control (Ajzen 1991).

“Attitude towards the behavior” refers to a person’s inclination to either favorably or unfavorably appraise the behavior of interest (Ajzen 1991), while “subjective norm” is defined as people’s estimation of the impact of social pressure on their decision to perform a behavior (Ajzen 1991). Perceived behavioral control, an addition to the original TRA, refers to “people’s perception of the ease or difficulty of performing the behavior of interest” (Ajzen 1991, p. 183).

The wide applicability of TPB is evidenced by its centrality in various researches into different forms of behavioral intention (Armitage and Conner 2001). Behavioral intentions, specifically the intention to produce contents for public consumption, in the online environment have also been increasingly studied using the theory. For instance, some (e.g., subjective norm) or all the TPB factors have been found to have significant effects on people’s intention to upload video contents (Park et al. 2011), post contents or share knowledge on a collaborative platform (Cho et al. 2010; Park et al. 2012), and post anonymous comments on a website (Soffer and Gordoni 2017). An extended version of TPB (with the inclusion of personality traits as predictors) was also tested in a study into people’s intention to review products online, although only one of the three TPB factors (attitude) predicted the intention of interest (Picazo-Vela et al. 2010).

Emerging from the results of the studies previously described is the first set of hypotheses.

Hypothesis 1: Mobile app users’ intention to write reviews for a mobile app is predicated on the three TPB factors, namely, (a) attitude towards writing reviews, (b) subjective norm, and (c) perceived behavioral control.

### The Functions of Online Review Writing and their Effects on Review Writing Intention

Daugherty et al. (2008) claim that people’s inclination to create user-generated contents (UGC), under which online reviews could be clustered, is anchored on four functions, namely, (a) utilitarian, (b) knowledge, (c) ego-defensive, and (d) value expressive. The authors argue that from a utilitarian perspective, the availability of incentives triggers UGC creation; whereas, from a knowledge standpoint, the need to understand themselves and their environment prompts people to create UGC. Moreover, Daugherty and colleagues noted that UGC creation based on an ego-defensive function is motivated by people’s need to minimize self-doubts, increase their sense of belongingness, and reduce feelings of guilt resulting from the decision not to contribute; on the other hand, UGC creation with a value-expressive motive, the authors added, is pursued to satiate one’s need for gratification feelings resulting from one’s contribution to a community and the need for validation of who they are.

In another empirical study into customers’ engagement in online word-of-mouth communication, which is practically similar to an online review (Chen and Xie 2008), it is reported that consumers’ disposition to publish their experiences with products on (online) opinion platforms is hinged on several considerations, namely, social benefits, economic incentives, concern for others, and self-enhancement (Hennig-Thurau et al. 2004). “Social benefits,” according to Hennig-Thurau et al., refer to product reviewers’ opportunity to interact with other customers, while “economic incentives” pertain to the availability of rewards for an individual decision to review products, which is remarkably similar to the utilitarian function of review writing.

The “concern for others” motive involves a personal need to notify potential customers of one’s positive and/or negative experiences with a product, while the “self-enhancement” motive refers to the psychological benefits one derives from being able to tell other customers about his or her product experience (Hennig-Thurau et al. 2004). The self-enhancement motive corresponds to Daugherty et al.’s ego-defensive function, while the concern for others motive appears to encompass both knowledge and value-expressive motives.

Therefore, based on these functions and motives, this research proposes that mobile app users’ intention to write review for a specific app is hinged on four critical functions, namely, (a) utilitarian, (b) social, (c) ego-defensive, and (d) emotional expression. The “emotional expression” is an extension of Daugherty et al.’s value-expressive motive and covers Hennig-Thurau et al.’s concern for others motive, as writing reviews predicated on the need to release one’s feelings towards an app may also serve the function of informing potential users of the app’s merits and flaws. From these points, the second set of hypotheses is proposed.

Hypothesis 2: Mobile app users’ intention to write reviews for a mobile app is predicated on four functions, namely, (a) utilitarian, (b) social, (c) ego-defensive, and (d) emotional expression.

### The Functions of Online Review Writing and their Effects on Attitude towards App Review Writing

A meta-analysis of various research using TPB reveals that of the three TBP factors hypothesized to influence behavior intention, attitude is a much better predictor of intention than subjective norm and perceived behavioral control (Armitage and Conner 2001). Such a finding reinforces the salient role of attitude as a determinant of intention, which subsequently implies that the factors that could strengthen attitude formation need identification.

Nowadays, a commonly held view on attitude is that it represents “an evaluative integration of cognitions and affects experience in relation to an object” (Crano and Prislin 2006, p. 347). The fact that attitude is a primary persuasion target (Bohner & Dickel, 2011; Crano and Prislin 2006; O’Keefe 2002) signifies that it can be changed through various means (O’Keefe 2002). In fact, previous studies have clearly shown that the attitude towards certain behaviors in the digital environment such as *softlifting* (illegal duplication of copyrighted software for personal use; Goles et al. 2008), online shopping (Chanaka 2004; Childers et al. 2001), and using travel-related user-generated contents (Ayeh et al. 2011) emerges from numerous intentionality-relevant factors such as perceived usefulness, enjoyment benefits, and trust.

Daugherty et al. (2008) also found that people’s attitude towards creating user-generated contents is predicated on the four functions (utilitarian, knowledge, ego-defensive, value expressive) of UGC creation. This point prompts the assumption that the functions of review writing do not only directly influence mobile app users’ intention to write reviews for apps but also their attitude towards app review writing. The third set of research hypotheses is, therefore, advanced.

Hypothesis 3: Mobile app users’ attitude towards writing reviews for mobile apps is predicated on the four functions of review writing, namely, (a) utilitarian, (b) social, (c) ego-defensive, and (d) emotional expression.

**Fig. 1:**
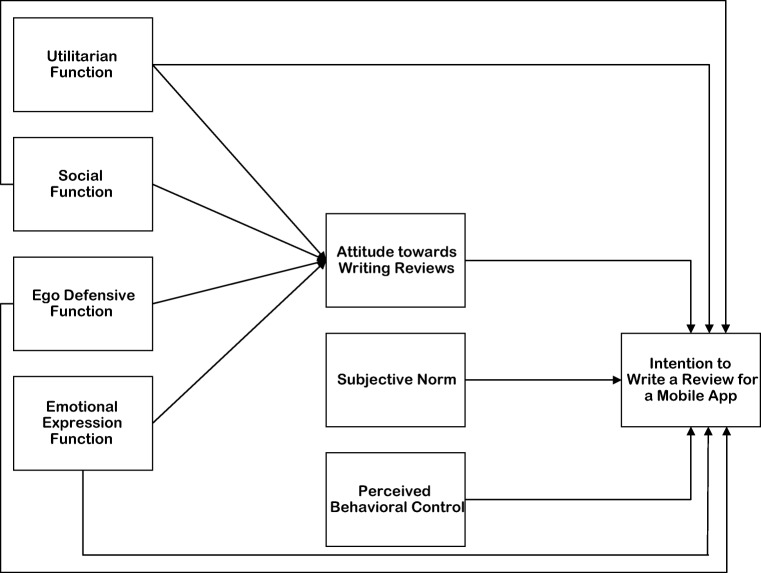
Research model for the determinants of intention to write a review for a mobile app and attitude towards writing reviews.

Figure 1 shows the complete research model that will be tested for this study.

## Research Method

### Research Design and Procedure

The hypotheses proposed for this study were tested with data collected from German mobile users through an online survey. To reach the intended respondents for the study, a snowball sampling technique was used, which involved sending an e-mail containing a link to the questionnaire to one of the researchers’ social networks. Recipients of the link were then requested to send it to individuals in their social networks.

The survey link was also posted on online social networking (OSN) sites and online forums to collect data from as many respondents as possible. More importantly, prior to the actual collection of survey data, the approval of the ethics committee of the university where the researchers are affiliated was secured.

For this study, the focus was only on a group of respondents who have already installed apps into the mobile phones since these are the individuals who are highly likely to write reviews for mobile apps. Based on a recent statistical data on smartphone penetration in Germany, there are 55,492,000 smartphone users in a country with a population of 80,636,000 (hence, a 68.8% smartphone penetration; Newzoo 2018).

### Research Participants

The several approaches used for data collection resulted in usable data from 203 German respondents. Calculation of the response rate was deemed impossible given the difficulty in determining the exact number of respondents who received the link to the survey.

In terms of respondents’ gender, a balance between male respondents (*n* = 97, 48%) and female respondents (*n* = 106, 52%) was almost achieved. Majority of the respondents belonged to the age cluster 18 and 34 (*n* = 168, 83%), with over half of the total number of survey respondents having a 4-year bachelor’s degree or higher (*n* = 133, 66%). Furthermore, in terms of mobile app type respondents regularly used, approximately 65% (*n* = 132) are frequent users of social networking apps. Presented on Table 1 is the complete demographic information of the survey respondents.

**Table 1:** Complete demographic information of research respondents

Variable	Categories	Frequency	Percent
*Gender*	Male	97	47.8
	Female	106	52.2
*Age*	18–24	55	27.1
	25–34	113	55.7
	35–44	23	11.3
	45 years or older	12	5.9
*Level of education*	Lower than high school	3	1.5
	High school education	10	4.9
	Some years in college	19	9.4
	Two-year professional education	37	18.2
	Four-year bachelor degree	77	37.9
	Graduate studies	56	27.6
	Others	1	0.5
*Type of mobile apps primarily used*	Entertainment/gaming	21	10.3
	Social networking	132	65.0
	Music	18	8.9
	Books/news/magazine	10	4.9
	Health and Fitness	7	3.4
	Shopping	3	1.5
	Banking	4	2.0
	Lifestyle	1	0.5
	Productivity	4	2.0
	Professional	3	1.5
*Total*		*203*	*100*

### Measurements

“Attitude toward writing reviews” was measured with three items on a five-point semantic differential scale. The items were derived from the scales of Daugherty et al. (2008) and Moon and Kim (2001). Three items were used to measure “perceived behavioral control,” two of which were originally formulated and another item a modified version of a statement by Netemeyer et al. (1991).

Subjective norm, as originally operationalized in TPB, has been criticized due to poor measurement, limited definition, and its inability to capture critical aspects of social influence (Armitage and Conner 2001). Although the concept originally refers to a person’s perception of social pressure to engage in a behavior (Ajzen 1991), social pressure is rarely considered direct or explicit (Armitage and Conner 2001).

Considering the rather individualistic nature of the decision to review a product, it is hardly the case that mobile users will seriously consider their significant others’ expectations and approval when deciding whether or not to write a review for an app. Instead, the decision would most likely be prompted by an awareness of what other people (within or outside one’s circle) are doing. Hence, subjective norm in this research is operationalized in terms of the appraised trendiness of the reviewing act as an impetus for a person’s disposition to write a review. Three items inspired by statements originally formulated by White et al. (2009) were used to measure subjective norm.

“Utilitarian function” was measured with four items that were substantially modified versions of the statements originally formulated by Daugherty et al. (2008). The remaining functions of reviewing—social, ego-defensive, and emotional expression—were measured with modified items based on the scales by Clary et al. (1994).

Four originally formulated items were used to measure the dependent variable “intention to write a review.” All the items (originally formulated in English and then translated to German) used for the different research constructs are presented on Table 2.

**Table 2:** Results of factor analysis with VARIMAX rotation of the items included in the online survey instrument (with mean and standard deviation values for the research constructs)

Constructs	Items	Factors						
		1	2	3	4	5	6	7
1. Utilitarian function (Daugherty et al. 2008)	(UTF1) I can win free app upgrades by writing an online review for a mobile app.				.78			
	(UTF2) Writing on online review for a mobile app is an opportunity to be virtually remunerated (e.g. in-app points, virtual money, etc.).				.73			
	(UTF3) By writing mobile app reviews I have the possibility to receive financial rewards.				.73			
	(UTF4) Writing a mobile app review offers me the possibility to earn free upgrades for that app.				.76			
2. Ego-defensive function (Clary et al. 1994)	(EGO1) Writing an online review for a mobile app makes me feel important.		.90					
	(EGO2) My self-esteem is increased when I write a review for a mobile app.		.87					
	(EGO 3) Writing an online review for a mobile app makes me feel needed.		.83					
3. Emotional expression function (Clary et al. 1994)	(EXP1) Writing a review enables me to express my frustration about the mobile app.	.86						
	(EXP2) Writing a review allows me to express my satisfaction with the app.	.88						
	(EXP3) When providing feedback for a mobile app, the review I submit reflects my thoughts and feelings about the app.	.76						
	(EXP4) Writing a review for a mobile app provides me with the opportunity to express my opinion about the app.	.86						
4. Attitude towards writing reviews (Daugherty et al. 2008; Moon and Kim 2001)	(ATT1) Writing a mobile app review is... pleasant/unpleasant.						.70	
	(ATT2) Writing a mobile app review is... good/bad.						.84	
	(ATT3) Writing a mobile app review is a... positive/negative thing.						.83	
5. Perceived behavioral control (*items* 1,3 *originally formulated*, *item 2 from* Netemeyer et al. 1991)	(PBC1) I have control over writing an online review for a mobile app.							.67
	(PBC2) For me, writing a mobile app review is easy.							.80
	(PBC3) If I wanted to, I could easily write a review for a mobile app.							.70
6. Subjective norm (*modified version of the items by* White et al. 2009)	(SUB1) A lot of people around me write mobile app reviews.					.87		
	(SUB2) A high percentage of people important to me write online reviews for mobile apps.					.84		
	(SUB3) I believe people around me provide feedback to app developers through reviews.					.80		
7. Intention to write reviews (*originally formulated items*)	(INT1) I will not hesitate writing reviews for mobile applications anytime soon.			.75				
	(INT2) I have a strong inclination to write a review for a mobile application in the coming weeks.			.76				
	(INT3) I do not see any problem in writing a review for a mobile application any time soon.			.57				
	(INT4) I will frequently submit reviews for mobile apps in the future.			.80				

### Measurement Validity and Reliability

To determine the constructs’ discriminant validity, items measuring both the independent and the dependent variables were subjected to a principal component analysis (PCA). The Kaiser-Meyer Olkin Measure of Sampling Adequacy was pegged at 0.79, higher than the recommended value of 0.60 (Kaiser 1974), while Bartlett’s Test of Sphericity [X^2^ (325) = 2469.87, *p* = .001] indicated that the correlations among the items were adequate for the performance of PCA.

Results of PCA indicate that the construct “social function” has a highly questionable validity, as the items selected to measure it loaded with “ego-defensive function” items. Merging the two sets of items was deemed inappropriate as they were supposed to measure two conceptually different constructs. This led to the decision to exclude social function from the research model, which means that hypotheses 2b and 3b could not be tested.

A second PCA was eventually performed, which resulted in a Kaiser-Meyer Olkin Measure of Sampling Adequacy value of 0.79 and a Bartlett’s Test of Sphericity value of [X^2^ (276) = 2247.54, *p* = .001]. The remaining constructs proved to have strong discriminant validity, as evidenced by the patterns of item loadings and the loading values. Table 2 shows the items used for various research constructs and the loading values for the different items.

Subsequently, structural equation modeling (SEM) technique using AMOS 22.0 was employed to test the model proposed for this research. In testing the research model, the two-step approach recommended by Anderson and Gerbing (1988), in which the measurement model was first assessed through confirmatory factor analysis (to determine the constructs’ convergent validity) prior to testing the research hypotheses with SEM, was used.

Based on the recommendations by Hu and Bentler (1999) and Schreiber et al. (2006), four indices were used to assess the fit of the measurement model and the full structural model: comparative fit index (CFI) and Tucker-Lewis index (TLI) to determine the model’s incremental fit (values for both CFI and TLI must be higher than .90; Hair et al. 2006), root-mean-square error of approximation (RMSEA) as a measure of absolute fit (RMSEA value must be lower than 0.08; Hair et al. 2006), and normed chi-square (X^2^*/df*), whose value must not exceed 5 for the model to be interpreted as acceptable (Wheaton et al. 1977).

Test of the measurement model indicates that it has an acceptable fit: *x*^2^ = 322.24, df = 209, *x*^2^/df = 1.54, *p* = .000, TLI = 0.93, CFI = 0.94, RMSEA = 0.05.

As both average variance extracted (AVE) and composite reliability (CR) values are good indicators of the constructs’ convergent validity, those values were also calculated. Recommended values for AVE and CR must be higher than 0.50 (Fornell and Larcker 1981) and 0.60 (Bagozzi and Yi 1988), respectively.

Table 3 presents the AVE and the CR values for the research constructs. Despite the acceptable CR values of UTF and PBC, their AVE values are below the cutoff point; hence, the two constructs’ convergent validity are deemed questionable.

**Table 3:** Convergent validity of the research constructs based item loading values, AVE values, and CR values

Construct	Items	Factor loadings	AVE	CR
Utilitarian function (UTF)	UTF 1	0.774	.440	.755
	UTF 2	0.579		
	UTF 3	0.563		
	UTF 4	0.714		
Ego-defensive function (EGO)	EGO1	0.905	.743	.896
	EGO2	0.824		
	EGO3	0.855		
Emotional expression function (EXP)	EXP1	0.835	.674	.891
	EXP2	0.897		
	EXP3	0.725		
	EXP4	0.816		
Attitude towards writing reviews (ATT)	ATT1	0.631	.564	.793
	ATT2	0.766		
	ATT3	0.840		
Subjective norm (SUB)	SUB1	0.861	.651	.841
	SUB2	0.853		
	SUB3	0.673		
Perceived behavioral control (PBC)	PBC1	0.589	.369	.637
	PBC2	0.603		
	PBC3	0.397		
Intention to write online reviews for mobile apps (INT)	INT1	0.749	.513	.806
	INT2	0.686		
	INT3	0.577		
	INT4	0.829		

## Results

### Test of the Original Model

Structural equation modeling was performed to test the original model proposed for this study. Test of the model indicates that its fit is not yet acceptable: *X*^2^ = 440.86, df = 240, X^2^/df = 1.84, *p* = .000, TLI = 0.89, CFI = 0.90, RMSEA = 0.06.

Regression estimates show that German app users’ intention to write reviews for a specific app is predicated on three factors, namely, a positive attitude towards review writing (*β* = 0.67, *p* < .001), social influence (*β* = 0.51, *p* < .001), and review writing’s expressive function (*β* = 0.28, *p* < .01). However, the hypothesized effects of review writing’s utilitarian (*β* = 0.10, *p* = .10) and ego-defensive functions (*β* = 0.17, *p* = .09) and perceived behavioral control (*β* = 0.06, *p* = .57) are not statistically significant.

Additionally, analysis indicates that German app users’ positive attitude towards review writing are anchored on two factors only, namely, ego-defensive function (β = 0.38, *p <* .001) and expressive function (β = 0.19, *p <* .05). The hypothesized effect of utilitarian function (*β* = 0.15, *p =* 11) on review writing intention, however, is not statistically significant.

### Test of the Modified Model

**Fig. 2:**
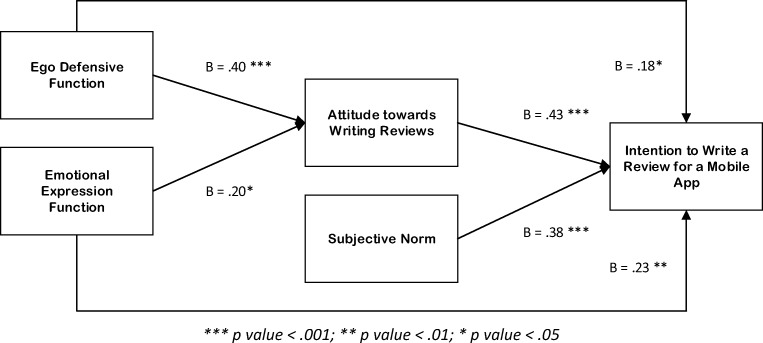
Modified version of a model for the factors influencing mobile app review writing intention. ***p < .001; **p < .01; *p < .05

Since the two constructs—utilitarian function and perceived behavioral control—do not have statistically significant effects on app review writing intention and have questionable convergent validity, as shown in Table 2, a modified version of the research model (Fig. 2), in which the two predictors were excluded, was consequently tested. The removal of the two predictors prompted a substantial improvement in model fit: *x*^2^ = 190.86, df = 109, *x*^2^/df = 1.75, *p* = .000, TLI = 0.94, CF = 0.95, RMSEA = 0.06.

The modification of the research model did not alter the results for the hypothesis testing segment of the study, as attitude (*β* = 0.43, *p <* .001), subjective norm (*β* = 0.38, *p <* .001), and the emotional expression function of review writing (*β* = 0.23, *p <* .01) remained significant predictors of review writing intention, despite a reduction in the paths’ beta values. Hence, hypotheses 1a, 1b, and 2d are supported, respectively.

What is worth noting, however, is that the removal of “attitude” and perceived behavioral control as predictors of “intention” resulted in ego-defensive function having a significant effect (*β* = 0.18, *p <* .05). This result subsequently supports hypothesis 2c.

Furthermore, the elimination of utilitarian function as a possible determinant of attitude did not alter the roles of both ego-defensive (*β* = 0.40, *p <* .001) and emotional expression (*β* = 0.20, *p <* .05) functions as predictors of attitude towards mobile app review writing. Thus, hypotheses 3c and 3d are also supported, respectively.

## Discussion of Results, Research Implications, and Future Research Directions

### Discussion of Results

App reviews, especially when their valence is positive, can serve as a low-cost marketing tool for an app. These could probably justify the drive on the part of app developers to persistently nudge users to write reviews for apps through various compensatory techniques (e.g., offering game points to users of game apps). However, to effectively stimulate app users to write reviews for certain apps, app developers ought to have insights into what would trigger users to write reviews.

Results of this study clearly indicate that app users’ intention to write reviews for mobile apps are predicated on two TPB factors, namely, attitude towards review writing and subjective norm. The fact that only two of three TPB factors play a role in people’s decision to review an app somehow confirms Ajzen’s (1991) assertion that the impact of these factors is bound to vary across behavioral intentions, behaviors, and situations.

Given the statistically insignificant effect of perceived behavior control on review writing intention, it can be assumed that the intention to perform the behavior of interest is not hinged on serious considerations of time availability and the ease of doing the act. For one, writing an app review is hardly a complex task that requires specialized skills, just as reviewing does not have to be a literary or journalistic endeavor demanding high-level writing aptitude and a sizable chunk of time. Moreover, the platform for review writing is relatively uncomplicated, which reduces the need to consult a voluminous user instruction guide.

What is apparent from the results, however, is that mobile users’ disposition to write reviews for certain apps is predicated on two factors, namely, a positive attitude towards the review writing act and an awareness of the extent to which individuals within one’s social environment write reviews. Mobile users will be inclined to review mobile apps when they regard the action as something positive, pleasant, or good. This seems indisputable given the impact of attitude on people’s propensity to perform various forms of behaviors across different contexts and situations.

Additionally, the pivotal role of subjective norm or social influence also merits attention considering the rather public component of the action’s consequence. Reviews are supposed to be written for public consumption; thus, the process of review writing has a strong public component making it possible for an individual to assess the intensity and the popularity of the act, as evidenced by the number of other individuals who have written reviews.

As previously mentioned, it is unrealistic to suppose that people’s decision to write a review would be based on their estimation of what their strong ties expect them to do, since review writing is not entirely an act whose performance is supposed to conform to societal norms. Unlike socially desirable actions with a strong ethical dimension (e.g., helping those in need or advocating for a social cause), review writing somehow lacks a strong moral dimension, unless one succumbs to the notion that reviewing an app is simply a moral thing to do. These points prompted the decision to re-conceptualize subjective norm not as an urge to conform to communal expectations but as an attempt to mimic the behavior of others.

A point that resonates from the results is that mobile app users would be predisposed to write reviews when others within their immediate social circles are doing the same. This finding is hardly new, as a recently published study also reported that people’s willingness to create user-generated contents for consumption in the environment (e.g., photos) is influenced by users’ awareness that individuals within their social groups also share (Beldad and Hegner 2017).

Results of the study additionally reveal that mobile app users’ intention to write reviews for apps is also predicated on two factors, namely, emotional expression and ego-defensive functions. The impact of emotional expression on the behavioral intention of interest should not come as a surprise since online reviews enable users to express their feelings towards a product or service purchased online (Folse et al. 2016; Kim and Gupta 2012).

Nonetheless, in this research, the emotional expression function is realized not only when one vents negative feelings towards an app or expresses for love for it, but also when that person aims at notifying potential users of an app’s merits and flaws. To a certain extent, then, the study’s finding partly affirms the point that online customers review products to display their concern for potential customers of the reviewed product (Hennig-Thurau et al. 2004).

The contribution of ego-defensive function to mobile users’ willingness to write reviews for apps also merits attention, as results of the current study show, further echoing findings of previous research (e.g., Daugherty et al. 2008; Hennig-Thurau et al. 2004). An implication of this result is that when people view the act of reviewing as something that could potentially enhance their feelings of relevance and self-worth, their willingness to write a review would consequently increase.

A positive attitude towards reviewing an app, results suggest, is based on two considerations, namely, the review writings’ ego-defensive and emotional expression functions. It has been reported in several studies that people’s attitude towards a specific action is predicated on a subjective appraisal of the action’s value and benefit. For instance, Daugherty et al. (2008) found that people’s attitude towards UGC creation is influenced by the ego-defensive and the social benefits that can be derived from the act.

Given the statistically insignificant effect of utilitarian function on people’s intention to write reviews for apps, one can surmise that mobile app users’ propensity to review apps could be an outcome of the calculative process they might have gone through. It is very likely that users would be prompted to review an app if the promised compensation for review writing is valued more highly than the effort and the time users have to invest in review writing. One can only assume that respondents for this study were not entirely enticed by the rewards they were offered when requested to review certain apps.

### Future Research Directions

Despite an initial attempt to propose and test an expanded version of the Theory of Planned Behavior to understand German mobile app users’ intention to write reviews for mobile apps, this research has not been spared from certain issues that have critical implications for the research results. Hence, results reported here must be cautiously interpreted.

The cross-sectional nature of this study could potentially limit claims pertaining to the causal relationships between the proposed predictors and people’s intention to write reviews for mobile apps. Future studies, therefore, should consider employing an experimental approach to test the possible impact of variables such as social influence and perceived psychological benefits on review writing intention.

Considering the research’s use of a small sample of German mobile users invited via a snowball sampling approach, the results of this study would hardly mirror the mechanisms behind app review writing among a wider population of German users. Furthermore, the use of data collected from a specific cultural or national cluster also limits the generalizability of research results to individuals belonging to other cultural/national clusters. The impact of the different factors on review writing intention would most likely vary across various cultural/national clusters.

Additionally, this study is also limited by its less nuanced view on app review writing intention across different categories of mobile apps (e.g., paid app vs free app, hedonistic app vs functional app). The results of this study would probably be different when the focus would be on a very specific type of mobile app, considering that variations in the experience of using a specific type of app might trigger variations in emotional responses, thereby resulting in variations in motivations for writing reviews for apps.

### Practical Implications

Results of this research have several implications for how mobile app designers can convincingly motivate app users to post reviews for certain apps. The pivotal role of attitude as a determinant of writing intention signifies the need for app developers to employ appropriate strategies to boost mobile users’ positive attitude towards review writing.

Based on results of this research, app developers can shape users’ attitude towards review writing by emphasizing the emotional expression and the ego-defensive benefits that can be derived from the writing act. One possible concrete action that could be taken is to underscore that reviewing app enables users not only to honestly express their views on the app but also to possibly notify potential users of an app’s merits and shortcomings. Framing the second point as a sort of helping behavior might appeal to the better senses of individuals who feel a strong urge to help others. Furthermore, positioning the review writing act as an ego-enhancing pursuit also has the potential to nudge users to positively view review writing.
